# Identification of Adipokine Clusters Related to Parameters of Fat Mass, Insulin Sensitivity and Inflammation

**DOI:** 10.1371/journal.pone.0099785

**Published:** 2014-06-26

**Authors:** Gesine Flehmig, Markus Scholz, Nora Klöting, Mathias Fasshauer, Anke Tönjes, Michael Stumvoll, Byung-Soo Youn, Matthias Blüher

**Affiliations:** 1 Department of Medicine, University of Leipzig, Leipzig, Germany; 2 Institute for Medical Informatics, Statistics and Epidemiology, University of Leipzig, Leipzig, Germany; 3 IFB ObesityDiseases, Junior Research Group Animal Models, University of Leipzig, Leipzig, Germany; 4 AdipoGen, College of Life Science and Biotechnology, Korea University, Seoul, Korea; 5 Immunomodulation Research Center, University of Ulsan, Ulsan, Korea; INSERM/UMR 1048, France

## Abstract

In obesity, elevated fat mass and ectopic fat accumulation are associated with changes in adipokine secretion, which may link obesity to inflammation and the development of insulin resistance. However, relationships among individual adipokines and between adipokines and parameters of obesity, glucose metabolism or inflammation are largely unknown. Serum concentrations of 20 adipokines were measured in 141 Caucasian obese men (n = 67) and women (n = 74) with a wide range of body weight, glycemia and insulin sensitivity. Unbiased, distance-based hierarchical cluster analyses were performed to recognize patterns among adipokines and their relationship with parameters of obesity, glucose metabolism, insulin sensitivity and inflammation. We identified two major adipokine clusters related to either (1) body fat mass and inflammation (leptin, ANGPTL3, DLL1, chemerin, Nampt, resistin) or insulin sensitivity/hyperglycemia, and lipid metabolism (vaspin, clusterin, glypican 4, progranulin, ANGPTL6, GPX3, RBP4, DLK1, SFRP5, BMP7, adiponectin, CTRP3 and 5, omentin). In addition, we found distinct adipokine clusters in subgroups of patients with or without type 2 diabetes (T2D). Logistic regression analyses revealed ANGPTL6, DLK1, Nampt and progranulin as strongest adipokine correlates of T2D in obese individuals. The panel of 20 adipokines predicted T2D compared to a combination of HbA1c, HOMA-IR and fasting plasma glucose with lower sensitivity (78% versus 91%) and specificity (76% versus 94%). Therefore, adipokine patterns may currently not be clinically useful for the diagnosis of metabolic diseases. Whether adipokine patterns are relevant for the predictive assessment of intervention outcomes needs to be further investigated.

## Introduction

The increasing incidence of obesity and type 2 diabetes leads to severe health consequences and financial burden of health systems. In most individuals, diagnosis of type 2 diabetes comes late, in an advanced state of associated complications with irreversible damages [1], [2].

Impaired adipose tissue function - one of the primary defects in obesity – is reflected by alterations in circulating adipokines which may link obesity to inflammation, insulin resistance and cardiovascular disease [3]–[6]. Adipokines are involved in various metabolic processes including the regulation of appetite control, satiety, energy expenditure, insulin sensitivity, inflammation, and cardiovascular function [7], [4]. Importantly, circulating adipokine patterns could be clinically relevant as markers of adipose tissue function and indicators of an increased metabolic risk [3], [8]. Adipose tissue secretes most likely more than 600 adipokines [9]. However, with the expanding number of newly identified adipokines there is an increasing need to define their function, molecular targets and potential clinical relevance in the treatment of obesity and metabolic diseases.

However, most previous studies have only investigated individual adipokines in defined human populations, whereas relationships among adipokines and between adipokines and parameters of obesity, glucose metabolism and inflammation are currently not well explored.

We therefore used an unbiased, distance-based hierarchical cluster analysis approach to recognize patterns among 20 adipokines and their relationship with parameters of fat mass, glucose metabolism, insulin sensitivity and inflammation in individuals with or without obesity-associated type 2 diabetes. In addition, we asked whether certain adipokine patterns may reflect obesity-associated type 2 diabetes better than established parameters (BMI, HbA1c, HOMA-IR). We selected these 20 adipokines based on the following criteria:

1. The molecule has been shown to be secreted from adipose tissue. For some of the analyzed adipokines we are aware that they are preferentially expressed in tissues other than adipose tissue (e.g. ANGPTL6, GPX3, NAMPT, progranulin, RBP4).
2. Relationships between adipokine serum concentrations and obesity, type 2 diabetes and/or adipose tissue function have been reported.
3. Availability of an adipokine assay, which has been internally validated in our research group.

In addition to adipokines for which associations between serum concentrations, obesity and metabolic traits have been well established (e.g. adiponectin, leptin, RBP4, resistin, omentin), we included several more recently identified adipokines (e.g. ANGPTL6, Clusterin, DLK1, DLL1, Glypican4, GPX3, SFRP5) whose links to obesity and obesity-related metabolic alterations are not commonly known or have not been studied in detail.

## Research design and Methods

### Study participants

A cross-sectional study was performed in 141 obese patients consecutively recruited in the context of a study on insulin resistance at the Department of Medicine, University of Leipzig. The study population included 67 Caucasian men and 74 women with a wide range of BMI, glycemia and insulin sensitivity (Table 1). The study protocol has been approved by the ethics committee of the University of Leipzig and conformed to the Declaration of Helsinki. Participants gave written informed consent before taking part in the study. Patients with severe conditions including generalized inflammation or advanced malignant diseases were excluded from the study. Individuals fulfilled the following inclusion criteria: 1) Absence of any acute or chronic inflammatory disease as determined by a leucocyte count > 14 Gpt/l, C-reactive protein (CrP) > 6.0 mg/dl or clinical signs of infection, 2) Undetectable antibodies against glutamic acid decarboxylase (GAD), 3) No clinical evidence of either cardiovascular or peripheral artery disease, 4) No thyroid dysfunction, 5) No alcohol or drug abuse, 6) No pregnancy.

**Table 1:** Anthropometric and clinical characteristics of the study population.

Parameter	Entire cohort	Obese, no T2D	Obese, T2D
N (women/men)	141 (74/67)	72 (39/33)	69 (35/34)
Age (years)	48±14	43±13	53±13*
	[19–80]	[19]–[66]	[29–80]
Antidiabetic pharmacotherapies:			
Metformin (n)	46	0	46
Sulfonylurea (n)	1	0	1
DPP-4 inhibitor (n)	4	0	4
GLP-1 analogues (n)	31	0	31
Pioglitazone (n)	3	0	3
Insulin (n)	12	0	12
BMI (kg/m^2^)	46.1±10.1	45.2±10.7	46.9±10.1
	[30.1–79.1]	[31.3–76.4]	[30.1–79.1]
Waist circumference (cm)	138±21	136±23	141±19
	[95–198]	[95–198]	[100–178]
Body fat (%)	42.1±8.4	42.0±7.7	42.0±9.2
	[22.3–65.9]	[24.8–57.3]	[22.3–65.9]
Fasting plasma glucose (mmol/l)	6.3±1.3	5.6±0.6	7.1±1.4*
	[4.6–10.7]	[4.6–7.5]	[4.7–10.7]
HbA1c (%)	6.4±1.0	5.7±0.3	7.1±1.0*
	[4.8–10.3]	[4. 8–6.5]	[5.9–10.3]
Fasting plasma insulin (pmol/l)	238±207	207±203	271±208*
	[16–1003]	[16–1003]	[28–843]
HOMA-IR	9.8±8.8	7.6±7.8	12.2±9.2*
	[0.6–45.1]	[0.6–36.1]	[1.1–45.1]
Triglycerides (mmol/l)	2.3±1.1	2.2±1.1	2.4±1.2
	[0.6–6.7]	[0.6–6.7]	[0.7–5.8]
HDL-Cholesterol (mmol/l)	1.1±0.2	1.2±0.2	1.1±0.3
	[0.6–1.8]	[0.7–1.8]	[0.6–1.7]
FFA (mmol/l)	0.64±0.27	0.61±0.29	0.67±0.25
	[0.13–1.53]	[0.13–1.53]	[0.22–1.47]
hsCRP (mg/dl)	0.96±0.94	0.87±0.97	1.06±0.92
	[0.06–5.62]	[0.06–5.62]	[0.11–4.52]
LPS (EU/ml)	11.5±5.2	11.2±4.3	11.7±6.0
	[4.8–39.9]	[4.8–31.0]	[6.3–39.9]
Adiponectin (µg/ml)	7.9±4.3	7.8±3.7	8.1±4.9
	[2.4–22.1]	[2.4–15.6]	[2.7–22.1]
ANGPTL3 (ng/ml)	114±46	113±50	115±43
	[33–261]	[33–258]	[45–261]
ANGPTL6 (ng/ml)	41.1±22.9	36.0±21.6	46.3±23.2*
	[6.6–113.1]	[6.8–98.7]	[6.6–113.1]
BMP7 (pg/ml)	25.6±15.2	25.4±16.0	26.0±14.4
	[6.5–109.5]	[6.5–107.5]	[11.2–109.5]
Chemerin (ng/ml)	238±57	232±60	245±54
	[96–390]	[96–390]	[140–380]
Clusterin (µg/ml)	59.2±16.0	58.8±14.8	59.6±17.4
	[25.0–109.8]	[31.7–97.1]	[25.0–109.8]
CTRP3 (ng/ml)	312±126	307±125	318±127
	[80–570]	[100–560]	[80–570]
CTRP5 (ng/ml)	68±23	70±27	66±20
	[29–199]	[29–199]	[30–147]
DLL1 (ng/ml)	29.4±9.6	29.0±10.0	29.9±9.2
	[11.1–56.4]	[13.4–56.4]	[11.1–54.5]
DLK1 (ng/ml)	4.0±4.3	4.6±4.2	3.4±4.3*
	[0.1–23.8]	[0.1–21.7]	[0.4–23.8]
Glypican4 (ng/ml)	7.3±6.2	8.0±6.8	6.6±5.3
	[1.0–37.2]	[1.7–37.2]	[1.0–36.6]
GPX3 (µg/ml)	3.23±0.82	3.15±0.91	3.32±0.72
	[1.76–5.65]	[1.76–5.65]	[1.91–5.09]
Leptin (ng/ml)	43.7±25.3	44.6±26.7	42.8±23.9
	[4.9–155.5]	[7.4–153.6]	[4.9–155.5]
NAMPT (ng/ml)	6.3±3.6	5.5±3.0	7.1±4.0*
	[0.5–17.9]	[1.4–17.9]	[0.5–16.5]
Omentin (ng/ml)	461±162	434±152	489±170*
	[147–941]	[147–907]	[166–941]
Progranulin (ng/ml)	208±55	196±55	222±52*
	[106–375]	[106–375]	[128–332]
RBP4 (µg/ml)	90.1±33.6	86.1±25.9	94.2±40.0
	[33.8–227.1]	[33.8–152.4]	[39.6–227.1]
Resistin (ng/ml)	10.3±3.8	10.2±3.5	10.4±4.1
	[2.1–23.6]	[3.6–23.6]	[2.1–21.6]
SFRP5 (ng/ml)	278±232	276±256	279±206
	[36–1288]	[36–1288]	[48–1067]
Vaspin (pg/ml)	754±736	792±746	714±729
	[110–4080]	[110–792]	[140–4080]

Clinical parameters and serum adipokine concentrations of 141 individuals and subgroups with or without type 2 diabetes (T2D). Data are means ± SD and [range]. *p value <0.05 for differences between obese patients with T2D and obese individuals without T2D.

Abbreviations: BMI, body mass index; HOMA-IR, Homeostatic Model Assessment – insulin resistance; FFA, free fatty acids; hsCRP, high sensitive C-reactive protein; LPS, Lipopolysacharid (Endotoxin); ANGPTL 3, angiopoietin-like protein 3; ANGPTL 6, angiopoietin-like protein 6; BMP7, bone morphogenetic protein 7; CTRP3, complement C1q tumor necrosis factor-related protein 3; CTRP5, complement C1q tumor necrosis factor-related protein 5; DLL1, delta-like protein 1; DLK1, preadipocyte factor 1; DPP-4, dipeptidyl peptidase-4; GPX3, glutathione peroxidase 3; NAMPT, nicotinamide phosphoribosyltransferase (visfatin); RBP4, retinol binding protein 4; SFRP5, secreted frizzled-related protein-5.

### Measurement of anthropometric and biochemical parameters

Body mass index (BMI) was calculated as weight divided by squared height. Waist circumference was measured at the midpoint between the lower ribs and iliac crest. Body fat was determined by Bio Impedance Analysis (Biacorpus RX 4000, Software: BodyComp V9.0, MEDI CAL HealthCare GmbH, Karlsruhe). All baseline blood samples were collected between 8:00 and 10:00am after an overnight fast. Fasting plasma insulin (FPI), fasting plasma glucose (FPG), HbA1c, high sensitivity C-reactive protein (hsCRP), high-density (HDL)-cholesterol, triglycerides (TG) and free fatty acids (FFA) were measured at the Institute of Laboratory Medicine, Clinical Chemistry and Molecular Diagnostics of the Universitätsklinikum Leipzig as previously described [8]. Insulin sensitivity was assessed using the Homeostatis model assessment – insulin resistance (HOMA-IR) index as ratio of FPI to FPG (FPI (pmol/l)/6.945 x FPG (mmol/l)/22.5). Lipopolysacharid (LPS, endotoxin) concentration was detected with a limuluis amebocyte lysate chromogenic endpoint assay (Hycultbiotech, Uden, The Netherlands).

### Measurement of adipokine serum concentrations

Serum concentrations of adipokines were detected by enzyme-linked immunosorbent assays (ELISA), sensitivity of each assay is specified in square brackets. Adiponectin [100 pg/ml], angiopoietin-like protein (ANGPTL) 3 [75 pg/ml], and 6 [1.2 ng/ml], complement C1q tumor necrosis factor-related protein (CTRP) 3 [1 ng/ml] and CTRP5 [1 ng/ml], delta-like protein 1 (DLL1) [120 pg/ml], preadipocyte factor 1 (DLK1) [336 pg/ml], glutathione peroxidase 3 (GPX3) [100 pg/ml], nicotinamide phosphoribosyltransferase or visfatin (NAMPT) [30 pg/ml], progranulin [32 pg/ml], retinol binding protein 4 (RBP4) [380 pg/ml], resistin [100 pg/ml] and vaspin [12 pg/ml] were measured using ELISAs (AdipoGen, Seoul, Korea). For measurement of leptin [0.2 ng/ml] and bone morphogenetic protein 7 - (BMP7) [4 pg/ml] serum concentrations, we used ELISAs of Mediagnost (Reutlingen, Germany). Serum concentrations of chemerin [0.1 ng/ml], clusterin [0.5 g/ml], and omentin [0.5 ng/ml] were determined by ELISA of Biovendor (Heidelberg, Germany). Serum levels of glypican4 [0.05 ng/ml], and secreted frizzled-related protein-5 (SFRP5) [0.58 ng/ml] were specified by ELISA of Uscn Life Science Inc. (Wuhan, China). The standard curve method with dilution series of provided human serum sample was used to generate a four parameter logistic curve-fit for determining the adipokine concentrations.

### Statistical analysis

Data are shown as means ± standard deviation unless stated otherwise. Data were tested for normal distribution by the Kolmogorov-Smirnov test. We compared two groups by t-test in data that were normally distributed and nonparametric Mann-Whitney U-test in non-normally distributed variables. To test dichotomous variables we run χ^2^ and Fisher exact tests. For clustering, we used Ward's minimum variance method and set a range of solutions from 2 to 5 clusters. We calculated approximately unbiased (AU) p-value and bootstrap probability (BP) value of the branching points of our cluster diagrams. AU p-values ≥ 90% were considered as strong evidence for the cluster. Analysis was performed using the function “pvclust” of the statistical software package “R” (www.r-project.org [10]). 10,000 bootstrapping replications were analysed.

In addition, we performed a principal components analysis (PCA) using serum adipokine concentrations and clinical parameters. The function “prcomp” of the statistical software package “R” (www.r-project.org) [10] was used for this analysis. Parameters were standardized prior to analysis and compared between T2D and no T2D applying U-tests. Plot of PCA and weights of features for the first two principal components are provided in Table S1 and Figure S1. Additionally, we performed a partial least square discriminant analysis (PLS-DA) applying an orthogonal scores algorithm as implemented in the function “plsr” of the statistical software package “R” (www.r-project.org) [10]. Parameters were standardized prior to analysis. We calculated VIP (variable importance in projection) to assess the importance of features regarding discrimination of T2D and no T2D. In supplement are plot of PLS-DA and VIP values provided. Prior to correlation and linear regression analysis, we excluded values >5 times of standard deviation. To test the predictive value of adipokines for type 2 diabetes state we used multivariate logistic regression analysis. Odds ratios were calculated with adjustment for age, sex, and BMI. Statistical analysis was performed using SPSS version 20.0 (Chicago, IL). P values <0.05 were considered to be statistically significant.

## Results

### Study population

We analyzed 67 men and 74 women with obesity defined as BMI ≥ 30 kg/m^2^. Among those, 72 individuals had a normal glucose metabolism and 69 were diagnosed with T2D (Table 1). Obese individuals are characterized by a wide range of anthropometric and glucose metabolism parameters (ranges of BMI: 30–79 kg/m^2^, waist circumference: 95–198 cm, body fat: 22–66%, HbA1c: 4.8–10.3%, FPG: 4.6–10.7 mmol/l, HOMA-IR: 0.6–45.1, FPI: 16–1003 pmol/l) (Table 1). Significant gender differences were detected for adiponectin, ANGPTL3, DLK1, and resistin with higher serum concentrations in women and for ANGPTL6, CTRP5, and RBP4 with higher levels in men (p<0.05; data not shown).

Mean BMI, BMI range and gender distributions were not different between individuals with or without type 2 diabetes (Table 1). However, patients with type 2 diabetes are significantly older compared to controls (Table 1). In addition to significant differences in glucose metabolism parameters (FPG, HbA1c, FPI, HOMA-IR) between T2D patients and controls, diagnosis of T2D was associated with significantly higher serum concentrations of ANGPTL6, Nampt, omentin, progranulin, as well as lower circulating DLK1 (Table 1). We further sought to determine, whether these and other differences in adipokine serum concentrations between individuals with and without T2D are due to concomitant anti-diabetic pharmacotherapy. T2D patients were treated with a variety of different medications including metformin, sulfonylurea, pioglitazone, DPP-4 inhibitors, GLP-1 analogs and insulin (Table 1). We found significant monotherapy effects associated with metformin on CTRP3 and Nampt and with insulin on circulating adiponectin, GPX3 and RBP4 (Table 2). Combination of metformin and GLP-1 analogs was associated with lower BMP7 serum concentrations compared to GLP-1 analog monotherapy (Table 2). Compared to insulin monotherapy, patients on metformin and insulin combination had significantly lower adiponectin and ANGPTL6 serum concentrations (Table 2). Interestingly, we did not find significant effects of sulfonylurea, DPP-4 inhibitor and pioglitazone treatment on any adipokine.

**Table 2:** Effects of different anti-diabetic pharmacotherapies on adipokine serum concentrations.

Adipokine	Metformin	no Metformin	p
N	46	23	
CTRP3 (ng/ml)	343±128	267±112	**0.018**
	[80–570]	[110–540]	
NAMPT (ng/ml)	6.5±3.9	8.3±4.0	**0.037**
	[0.8–16.5]	[0.5–15.3]	
	**Insulin**	**no Insulin**	
N	12	57	
Adiponectin (µg/ml)	5.9±4.1	8.6±5.0	**0.018**
	[2.8–16.9]	[2.7–22.1]	
GPX3 (µg/ml)	3.76±0.57	3.22±0.71	**0.018**
	[3.04–5.09]	[1.91–5.04]	
RBP4 (µg/ml)	72.9±20.8	98.7±41.5	**0.040**
	[46.2–113.6]	[39.6–227.1]	
	**Metformin + GLP-1 analogue**	**GLP-1 analogue monotherapy**	
N	29	40	
BMP7 (pg/ml)	30.8±18.8	22.4±8.9	**0.019**
	[12.7–109.5]	[11.2–60.3]	
	**Metformin + Insulin**	**Insulin monotherapy**	
N	8	61	
Adiponectin (µg/ml)	5.7±4.7	8.4±4.9	**0.016**
	[2.8–16.9]	[2.7–22.1]	
ANGPTL3 (ng/ml)	85±10	119±43	**0.012**
	[70–99]	[45–261]	

Comparison of adipokine serum concentrations in patients treated with or without metformin in monotherapy or in combination with GLP-1 analogues and insulin, as well as insulin monotherapy. Only significant differences between the different treatment regimens are displayed. Treatment with sulfonylurea, pioglitazone or DPP-4 inhibitors did not significantly affect serum adipokine concentrations.

### Adipokine clusters related to parameters of fat mass, inflammation or insulin sensitivity

We used an unbiased approach to recognize relationships among adipokines and between adipokines and established clinical parameters of obesity (BMI, waist circumference, body fat), inflammation (hsCrP, LPS), glucose metabolism (HbA1c, HOMA-IR), and lipid metabolism (HDL-cholesterol, TG and FFA). We investigated a total of 30 variables, including 20 adipokines (adiponectin, ANGPTL3, ANGPTL6, BMP7, chemerin, clusterin, CTRP3, CTRP5, DLL1, DLK1, glypican 4, GPX3, leptin, NAMPT, omentin, progranulin, RBP4, resistin, SFRP5, vaspin).

**Figure 1:**
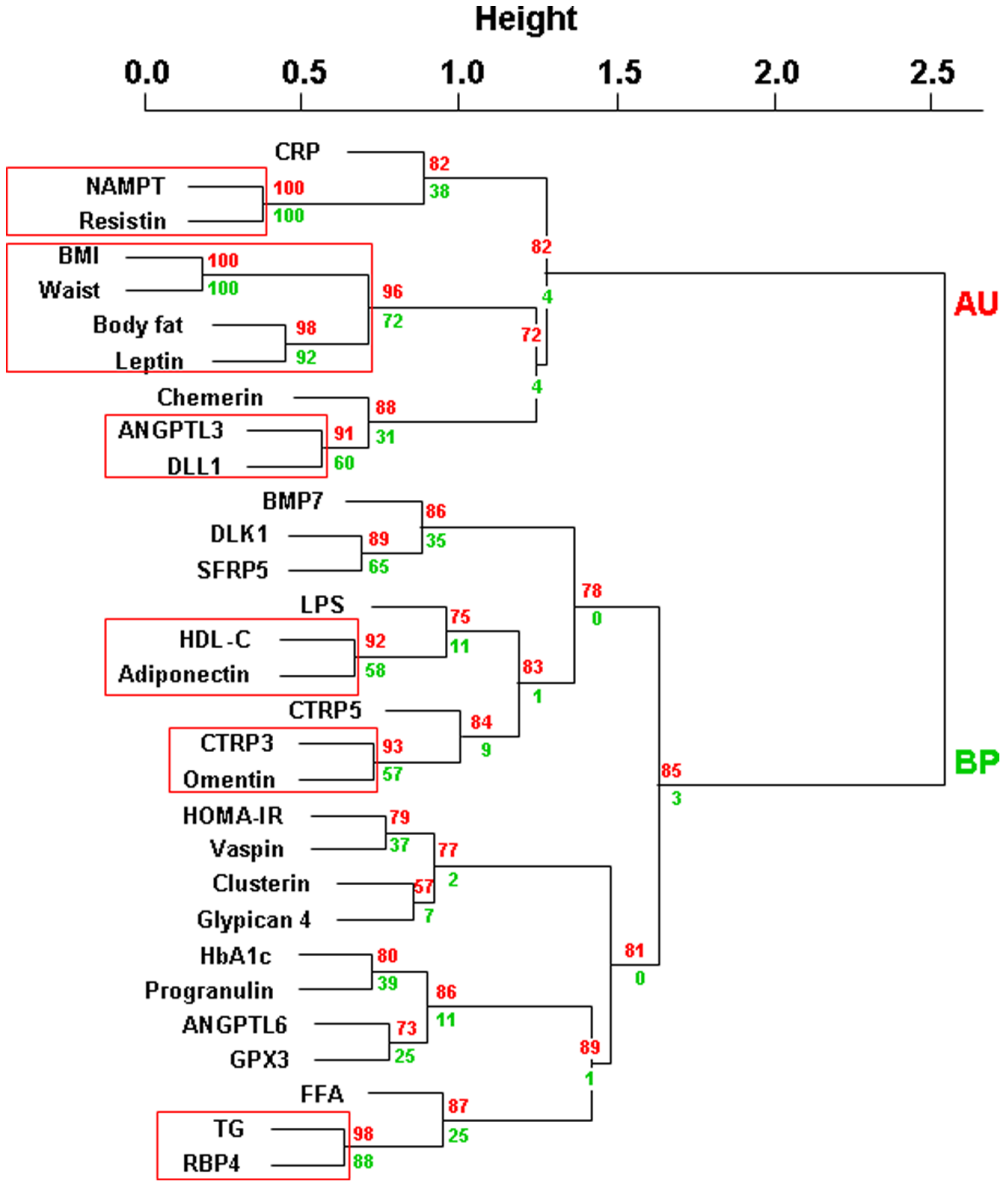
Hierarchical clustering of serum adipokine concentrations and clinical parameters in obese patients (n = 141). We provide approximately unbiased (AU, numbers in red) p-values and bootstrap probability (numbers in green) values as measures of certainty for clusters. Values are based on 10,000 bootstrapping replicates. AU≥90% was considered as strong evidence for the cluster and is marked by red rectangles (only largest possible clusters are marked). Abbreviations: BMI, body mass index; HOMA-IR, Homeostatic Model Assessment; FFA, free fatty acids; CRP, high sensitive C-reactive protein; ANGPTL 3, angiopoietin-like protein 3; ANGPTL 6, angiopoietin-like protein 6; BMP7, bone morphogenetic protein 7; CTRP3, complement C1q tumor necrosis factor-related protein 3; CTRP5, complement C1q tumor necrosis factor-related protein 5; LPS, Lipopolysacharid (Endotoxin); GPX3, glutathione peroxidase 3; DLL1, delta-like protein 1; DLK1, preadipocyte factor 1; NAMPT, nicotinamide phosphoribosyltransferase (visfatin); RBP4, retinol binding protein 4; SFRP5, secreted frizzled-related protein-5; TG, triglycerides

Analysis of these 30 variables in the entire study cohort (n = 141) revealed 2 main clusters, which are most closely related to either body fat mass and inflammation (leptin, ANGPTL3, DLL1, chemerin, Nampt, resistin) or insulin sensitivity/hyperglycemia, and lipid metabolism (adiponectin, vaspin, clusterin, glypican 4, progranulin, ANGPTL6, GPX3, RBP4, DLK1, SFRP5, BMP7, CTRP3 and 5, omentin) (Figure 1). As an internal validation of the model, we confirmed significant and robust closest relationships between BMI and waist as well as leptin and body fat mass [11] (Figure 1). Approximately unbiased p-values and bootstrap probability values were used as measures of certainty for clusters and all values are based on 10,000 bootstrapping replicates. We considered approximately unbiased p-values ≥ 90% as strong evidence for a cluster. Using this criterion, we identified six clusters of parameters, which are most closely related including NAMPT and resistin, BMI, waist, body fat mass and leptin (internal model validation), ANGPTL3 and DLL1, HDL-cholesterol and adiponectin, CTRP3 and omentin, triglycerides and RBP4 (Figure 1).

**Figure 2:**
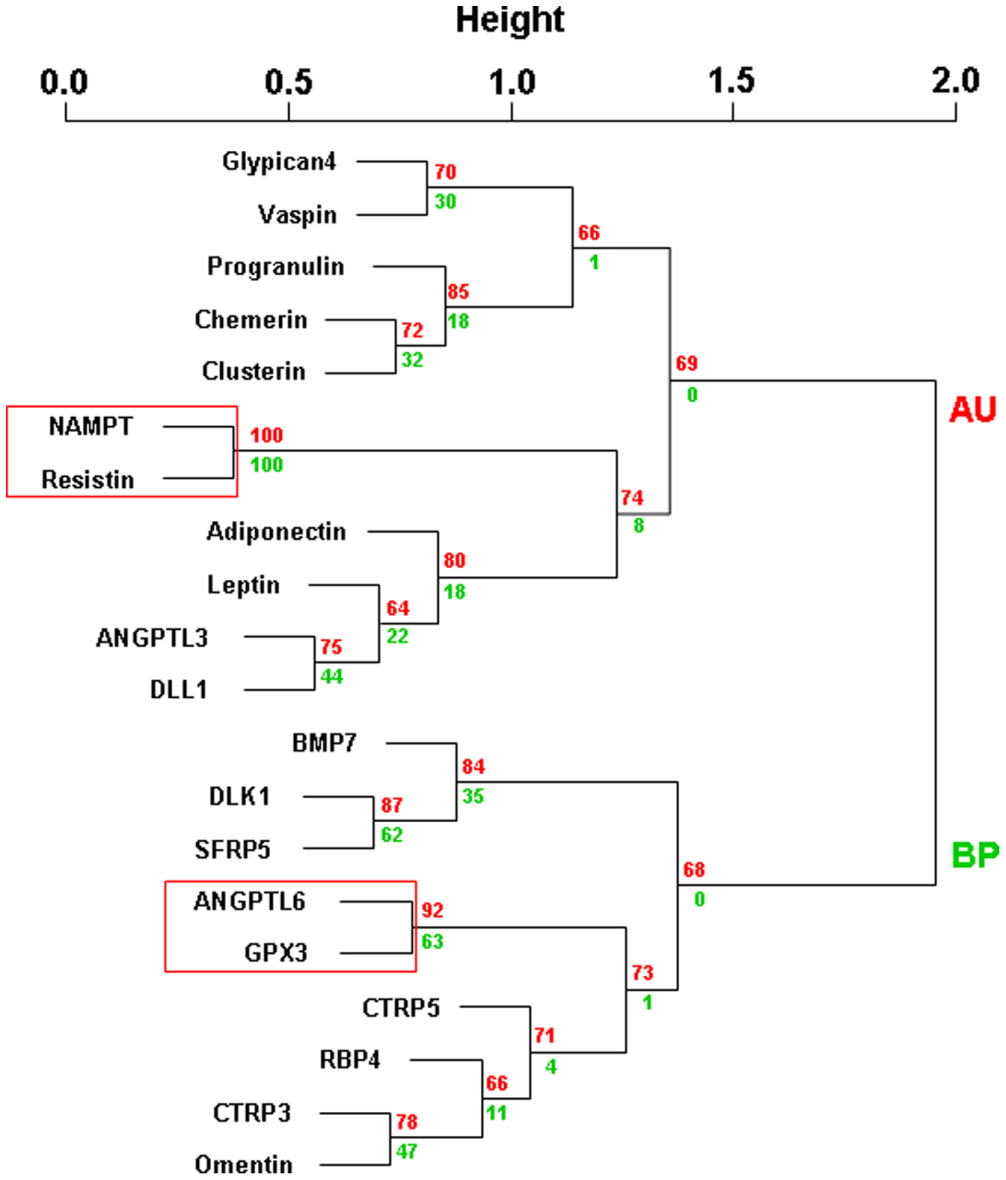
Hierarchical clustering of 20 serum adipokine concentrations in obese patients (n = 141). We provide approximately unbiased (AU, numbers in red) p-values and bootstrap probability (numbers in green) values as measures of certainty for clusters. Values are based on 10,000 bootstrapping replicates. AU≥90% was considered as strong evidence for the cluster and is marked by red rectangles (only largest possible clusters are marked). Abbreviations: CRP, high sensitive C-reactive protein; ANGPTL 3, angiopoietin-like protein 3; ANGPTL 6, angiopoietin-like protein 6; BMP7, bone morphogenetic protein 7; CTRP3, complement C1q tumor necrosis factor-related protein 3; CTRP5, complement C1q tumor necrosis factor-related protein 5; LPS, Lipopolysacharid (Endotoxin); GPX3, glutathione peroxidase 3; DLL1, delta-like protein 1; DLK1, preadipocyte factor 1; NAMPT, nicotinamide phosphoribosyltransferase (visfatin); RBP4, retinol binding protein 4; SFRP5, secreted frizzled-related protein-5; TG, triglycerides.

In order to exclude clustering effects of obvious associations (e.g. between BMI, waist, body fat mass and leptin) on adipokine relationships, we performed additional cluster analysis for the 20 adipokines only (Figure 2). Using the same stringent criteria as strong evidence for a cluster, only the relationships between NAMPT and resistin and the relationship between ANGPTL6 and GPX3 can be considered significant (Figure 2).

### Adipokine clusters in patients with or without type 2 diabetes

**Figure 3:**
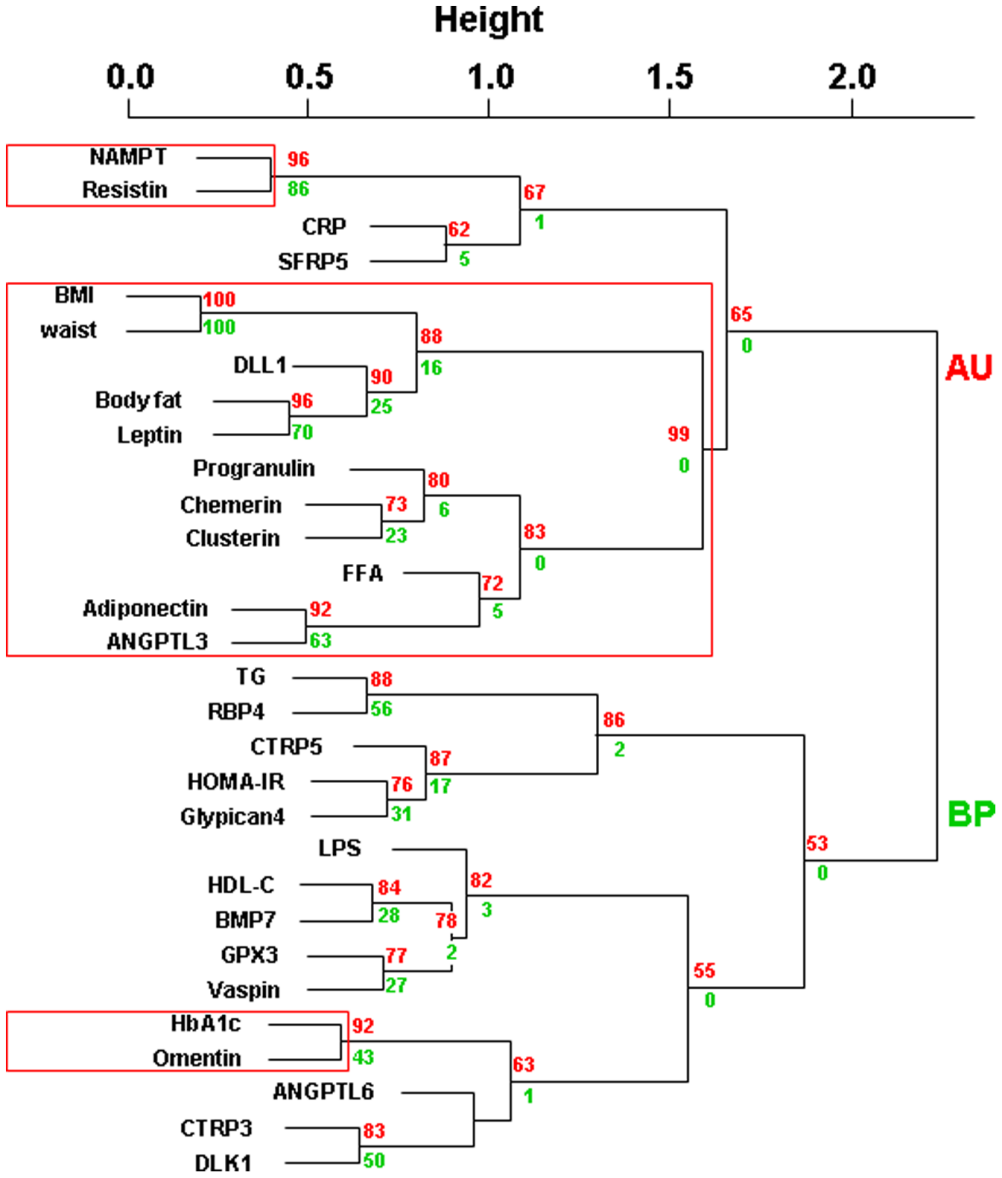
Hierarchical clustering of serum adipokine concentrations and clinical parameters in controls without type 2 diabetes (n = 72). We provide approximately unbiased (AU) p-values and bootstrap probability (BP) values as measures of certainty for clusters. Values are based on 10,000 bootstrapping replicates. AU≥90% was considered as strong evidence for the cluster and is marked by red rectangles (only largest possible clusters are marked). Abbreviations: BMI, body mass index; HOMA-IR, Homeostatic Model Assessment; FFA, free fatty acids; CRP, high sensitive C-reactive protein; ANGPTL 3, angiopoietin-like protein 3; ANGPTL 6, angiopoietin-like protein 6; BMP7, bone morphogenetic protein 7; CTRP3, complement C1q tumor necrosis factor-related protein 3; CTRP5, complement C1q tumor necrosis factor-related protein 5; LPS, Lipopolysacharid (Endotoxin); GPX3, glutathione peroxidase 3; DLL1, delta-like protein 1; DLK1, preadipocyte factor 1; NAMPT, nicotinamide phosphoribosyltransferase (visfatin); RBP4, retinol binding protein 4; SFRP5, secreted frizzled-related protein-5; TG, triglycerides.

**Figure 4:**
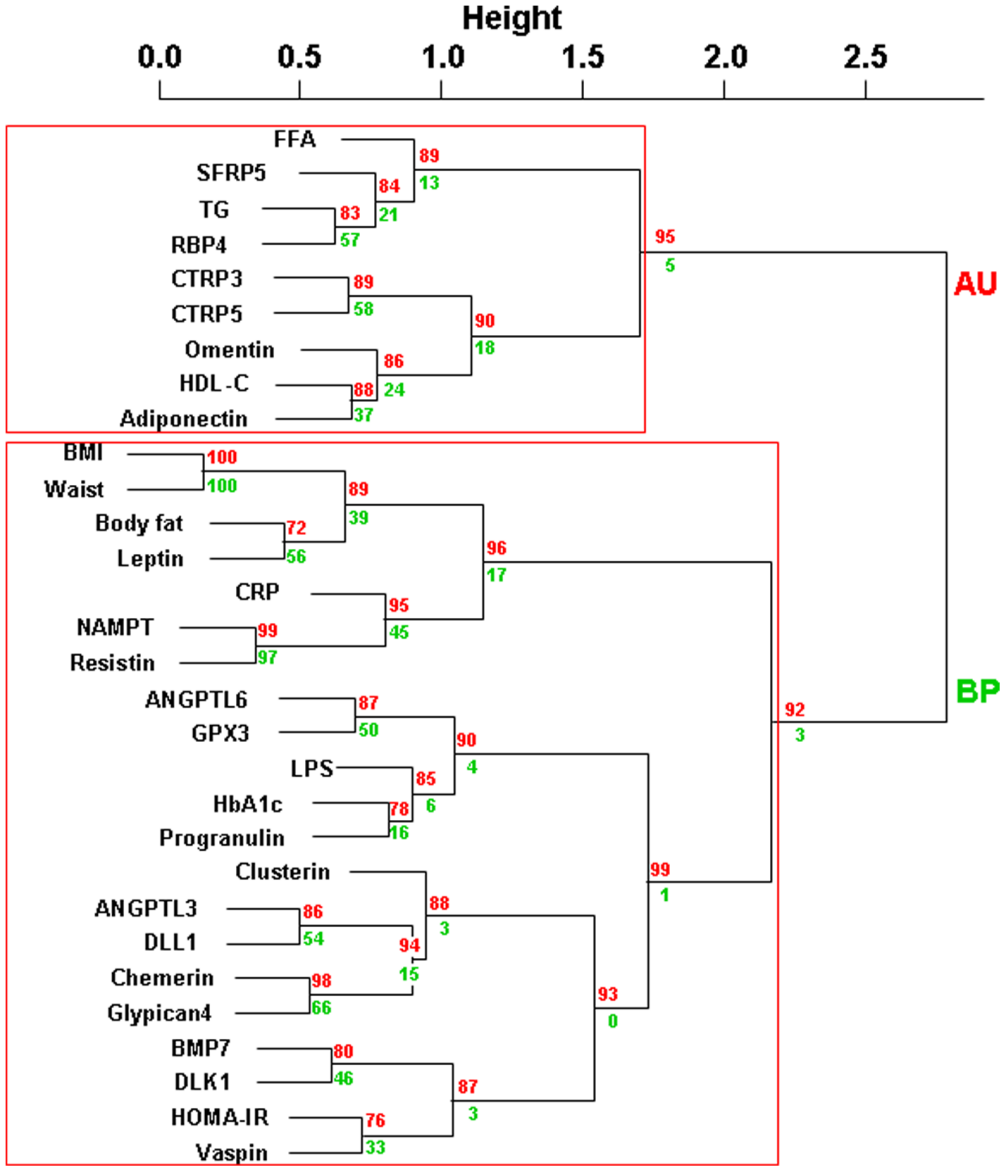
Hierarchical clustering of serum adipokine concentrations and clinical parameters in patients with type 2 diabetes (n = 69). We provide approximately unbiased (AU) p-values and bootstrap probability (BP) values as measures of certainty for clusters. Values are based on 10,000 bootstrapping replicates. AU≥90% was considered as strong evidence for the cluster and is marked by red rectangles (only largest possible clusters are marked). Abbreviations: BMI, body mass index; HOMA-IR, Homeostatic Model Assessment; FFA, free fatty acids; CRP, high sensitive C-reactive protein; ANGPTL 3, angiopoietin-like protein 3; ANGPTL 6, angiopoietin-like protein 6; BMP7, bone morphogenetic protein 7; CTRP3, complement C1q tumor necrosis factor-related protein 3; CTRP5, complement C1q tumor necrosis factor-related protein 5; LPS, Lipopolysacharid (Endotoxin); GPX3, glutathione peroxidase 3; DLL1, delta-like protein 1; DLK1, preadipocyte factor 1; NAMPT, nicotinamide phosphoribosyltransferase (visfatin); RBP4, retinol binding protein 4; SFRP5, secreted frizzled-related protein-5; TG, triglycerides.

We divided the entire study group of obese individuals into those with or without T2D (Table 1) (Figure 3). Individuals with or without T2D did not differ with regard to anthropometric parameters, but had significantly different levels of FPG, FPI, HbA1c, and HOMA-IR (Table 1). Cluster analysis of individuals with T2D shows different cluster consistence and closest relationships compared to individuals without T2D (Figures 3 and 4). Consistent between obese individuals with or without T2D, we find closest relationships between NAMPT and resistin, RBP4 and triglycerides and as a proof of validity of the model between BMI and waist as well as between leptin and body fat mass [11] (Figures 3 and 4). The detection of these well established relationships by unbiased cluster analyses suggests that other previously unrecognized relationships (e.g. NAMPT and resistin) are not randomly identified.

In obese individuals with normal glucose metabolism, we find significant clusters of omentin and HbA1c, NAMPT and resistin as well as a larger (anthropometric parameter driven) cluster containing leptin, DLL1, progranulin, chemerin, clusterin, adiponectin and ANGPTL3 (Figure 3). Interestingly, in patients with T2D, parameters included into the analyses dissociate into two significant main clusters, which either reflect lipid metabolism (TG, FFA, HDL-cholesterol) or a combination of glucose metabolism, anthropometric and inflammation parameters (Figure 4). Adiponectin, omentin, CTRP3 and 5, RBP4 and SFRP5 cluster with lipid metabolism parameters, whereas all other adipokines are in the larger cluster of BMI, hsCrP, HbA1c and HOMA-IR (Figure 4). This may suggest that under conditions of T2D and obesity, adiponectin, omentin, CTRP3 and 5, RBP4 and SFRP5 reflect additional alterations in lipid metabolism. Moreover, these adipokines may help to identify subgroups of obese patients with T2D and additional disturbances of lipid metabolism.

### Do adipokine patterns distinguish obese individuals with or without T2D?

To determine whether and which adipokine serum concentrations may detect patients with T2D among obese individuals (n = 141), we performed multivariate logistic regression analysis and principle component analyses. In the first multivariate model for the prediction of T2D, we included all 20 adipokines together with age, gender and BMI (Table 3). For model 1, sensitivity to detect T2D is 78.3% with a specificity of 76.4% and a correct prediction 76.6% of the time overall (Table 4). Model 2 describes the strongest predictors of T2D identified in the last step of a Wald stepwise backwards analysis. In addition to age, an adipokine pattern of ANGPTL6, DLK1, Nampt, and progranulin significantly predicts T2D (Table 3). Using these variables, we found a predictive value for T2D with true positive rate of 65.2%, true negative rate of 73.6% and prediction accuracy of 69.5% (Table 4). We confirmed these data by principal component analysis (PCA) supporting the significant predictive value of age, ANGPTL6, DLK1, NAMPT, and progranulin for T2D (Table S1, figure S1). Further supporting the validity of these analyses, PCA identified HbA1c and HOMA-IR as significant variables in the prediction of T2D (Table S1). To further support variables identified in model 2 of the multivariate logistic regression analysis, we performed parameters of partial least square discriminant analysis (PLS-DA) and calculated VIP values to assess the weight of parameters discriminating obese patients with or without T2D (Table S2, figure S2). Importantly, these analyses confirmed the variables (age, ANGPTL6, NAMPT, progranulin) identified in multivariate logistic regression analysis models (Table 3) except DLK1. However, compared to these adipokine-based predictors of T2D, a combination of classical clinical parameters including HbA1c, FPG and HOMA-IR (all adjusted for BMI, age and gender) predict T2D with a sensitivity of 91.3% and a specificity of 94.4% in our study population (Table 4).

**Table 3:** Multivariate-adjusted odds ratios of adipokines as predictors of type 2 diabetes in obese individuals (n = 141).

Model 1	B	SE	OR (95% CI)	p-value	r^2^ = 0.477
Adiponectin	−.055	.064	0.95 (0.84–1.08)	0.388	
ANGPTL3	−.004	.006	1.00 (0.98–1.01)	0.493	
ANGPTL6	.018	.012	1.02 (0.99–1.04)	0.140	
BMP7	.021	.015	1.02 (0.99–1.05)	0.165	
Chemerin	.006	.005	1.01 (1.00–1.02)	0.269	
Clusterin	−.009	.019	0.99 (0.96–1.03)	0.631	
CTRP3	.004	.002	1.00 (1.00–1.01)	0.104	
CTRP5	.004	.010	1.00 (0.98–1.03)	0.673	
DLL1	.010	.030	1.01 (0.95–1.07)	0.737	
DLK1	−.227	.071	0.80 (0.70–0.92)	0.001	
Glypican4	−.063	.041	0.94 (0.87–1.02)	0.124	
GPX3	−.270	.295	0.76 (0.43–1.36)	0.359	
Leptin	−.011	.012	0.99 (0.97–1.01)	0.374	
Nampt	.320	.102	1.38 (1.13–1.68)	0.002	
Omentin	.000	.002	1.00 (1.00–1.00)	0.826	
Progranulin	.011	.004	1.01 (1.00–1.02)	0.014	
RBP4	.006	.009	1.01 (0.99–1.02)	0.492	
Resistin	−.071	.096	0.93 (0.77–1.12)	0.457	
SFRP5	.001	.001	1.00 (1.00–1.00)	0.638	
Vaspin	.000	.000	1.00 (1.00–1.00)	0.306	
gender	.604	.532	1.8 (0.65–5.19)	0.256	
age	.096	.026	1.10 (1.05–1.16)	<0.0001	
BMI	.047	.035	1.05 (0.98–1.12)	0.182	
Constant	−11.965	3.402		<0.0001	
Model 2		SE	OR (95% CI)	p-value	r^2^ = 0.389
ANGPTL6	.025	0.010	1.03 (1.01–1.05)	0.016	
DLK1	−.132	0.051	0.88 (0.79–0.97)	0.011	
Nampt	.221	0.065	1.25 (1.10–1.42)	0.001	
Progranulin	.008	0.004	1.01 (1.00–1.02)	0.037	
age	.073	0.018	1.08 (1.04–1.11)	<0.0001	
Constant	−7.101	1.414		0.001	

Logistic regression analysis: regression coefficients (B), standard errors (SE), odds ratios (OR), p-values and Nagelkerke r^2^ for prediction models of T2D in 141 obese individuals. Model 1 includes all 20 adipokines, model 2 shows the parameters with the strongest predictive value for T2D as result of a backwards stepwise method. Abbreviations: BMI, body mass index; ANGPTL 3, angiopoietin-like protein 3; ANGPTL 6, angiopoietin-like protein 6; BMP7, bone morphogenetic protein 7; CTRP3, complement C1q tumor necrosis factor-related protein 3; CTRP5, complement C1q tumor necrosis factor-related protein 5; DLL1, delta-like protein 1; DLK1, preadipocyte factor 1; GPX3, glutathione peroxidase 3; Nampt, nicotinamide phosphoribosyltransferase (visfatin); RBP4, retinol binding protein 4; SFRP5, secreted frizzled-related protein-5.

**Table 4:** Sensitivity and specificity of adipokines and clinical parameters for T2D among obese.

Model	Variables	Sensitivity (%)	Specificity (%)	Prediction accuracy (%)
[1]	20 adipokines +BMI, age, gender	78.3	76.4	77.3
[2]	ANGPTL6, DLK1, Nampt progranulin + age	65.2	73.6	69.5
[3]	HbA1c, FPG, HOMA-IR + BMI, age gender	91.3	94.4	92.9

Logistic regression analysis: Sensitivity, specificity and prediction accuracy for T2D in 141 obese individuals. Model 1 includes all 20 adipokines, whereas model 2 represents parameters with the strongest predictive value for T2D identified by Wald backwards stepwise test. All models are adjusted for BMI, age and gender.

Abbreviations: BMI. body mass index; FPG, fasting plasma glucose; HOMA_IR, Homeostatic Model Assessment; ANGPTL 6, angiopoietin-like protein 6; DLK1, preadipocyte factor 1; Nampt, nicotinamide phosphoribosyltransferase (visfatin).

## Discussion

Adipose tissue is a highly active endocrine organ secreting a number of bioactive molecules (adipokines). Altered adipokine secretion can be considered as a symptom of adipose tissue dysfunction [12]. The search for novel adipokines linking obesity to related co-morbidities has become a major topic in obesity research [7]. However, with the expanding number of newly identified adipokines, there is an increasing need to define their function, molecular targets and potential clinical relevance as biomarkers or in the treatment of obesity and metabolic diseases. In addition, relationships among adipokines and between adipokines and anthropometric and biochemical parameters need to be elucidated.

We therefore used an unbiased approach (unbiased, distance-based hierarchical cluster analysis) to recognize adipokine patterns associated with parameters of obesity, glucose metabolism, insulin sensitivity and inflammation.

Here, we identified two major adipokine clusters related to either body fat mass/inflammation (leptin, ANGPTL3, DLL1, chemerin, Nampt, resistin) or insulin sensitivity/hyperglycemia/lipid metabolism (adiponectin, vaspin, clusterin, glypican 4, progranulin, ANGPTL6, GPX3, RBP4, DLK1, SFRP5, BMP7, CTRP3 and 5, omentin).We consider the detection of well-established relationships such as between BMI and waist circumference, leptin and body fat mass [11] or TG and FFA serum concentrations as proof of the internal data and analyses quality.

As an example, we confirm previously reported close relationships between circulating RBP4 and serum triglycerides [13], [14]. For RBP4, relationships with insulin resistance and glucose homeostasis have been controversially discussed [14], [15]–[17]. Our data suggest that previously reported associations between RBP4 and insulin resistance [15] may be influenced by triglyceride and FFA serum concentrations.

The cluster of adipokines and insulin sensitivity/hyperglycemia/lipid metabolism parameters is consistent with reported associations between markers of insulin sensitivity, glucose homeostasis and adiponectin [18], [19], DLK1 [20], SFRP5 [21], vaspin [22], glypican4 [23], CTRP3 [24] and omentin [25]. Interestingly, most of the adipokines (adiponectin, vaspin, SFRP5, omentin, glypican4) in this cluster have been previously linked to adipose tissue dysfunction, adipocyte hypertrophy and adverse (visceral) fat distribution [8], [17], [20], [23], [26]–[30]. This supports the hypothesis that adipose tissue dysfunction contributes to obesity related metabolic diseases [6]. In addition, our cluster analyses may have identified previously unrecognized associations between circulating BMP7 and CTRP5 with insulin sensitivity and glucose metabolism.

For several adipokines (ANGPTL3, DLL1, chemerin, clusterin, leptin, Nampt, resistin, GPX3), we identified a close relationship with parameters of obesity (BMI, waist circumference, body fat mass), but also inflammation (hsCrP). In accordance with that, associations with both body weight and inflammation have been reported for chemerin [31], [32], clusterin [33], leptin [11], Nampt [34], resistin [35]. Since ANGPTL3, DLL1 and GPX3 are found in the same cluster, our data may stimulate further research characterizing the potential role of ANGPTL3, DLL1 and GPX3 in obesity, altered glucose metabolism and inflammation. The close relationship between inflammation and impaired insulin sensitivity is well established [36]. A limitation of our cluster analyses is that inflammation was only characterized by circulating hsCRP and that only individuals with normal hsCrP have been included. We can therefore not exclude that a wider range of hsCrP as well as inclusion of additional markers of inflammation, such as TNFα, interleukin 6, plasminogen activator inhibitor-1 (PAI-1), transforming growth factor β (TGF β) or monocyte chemotactic protein-1 (MCP-1) may have identified different adipokine clusters related to inflammation. Nevertheless, in the Framingham study only hsCRP was significantly associated with insulin resistance among 9 different inflammation markers [37].

Cluster analyses of adipokines only (Figure 2) revealed significant relationships beyond those identified in analyses containing additional parameters. Consistent relationships were found for NAMPT and resistin as well as for ANGPTL6 and GPX3. Further studies are required to identify potential mechanistic links between these and other related (e.g. glypican4 and vaspin, DLK1 and SFRP5) pairs of adipokines. Importantly, most of the relationships we find between adipokines have not been reported before and should be further analyzed in subsequent studies.

Early identification of pathogenic factors in the development of obesity related metabolic diseases such as insulin resistance, chronic inflammation, insulin secretion defect or adipose tissue dysfunction could result in personalized prevention or treatment strategies for individuals at high risk. Since treatment of obesity and prevention of obesity related diseases is an enormous medical and socio-economic task which will not always be successful, it is important to define the obese patient who will benefit the most from early lifestyle, but also bariatric surgery or pharmacological interventions. For the stratification of obesity treatment, definition of metabolically healthy or high-risk phenotypes will facilitate the identification of the obese person who requires early and more intensive treatment strategies. However, there is an unmet need for biomarkers, which may help to distinguish metabolically healthy from high risk obese individuals. In this context, we previously identified adiponectin, progranulin, chemerin, Fetuin-A and RBP4 serum concentrations as significant predictors of insulin sensitive or metabolically healthy obesity [8]. Although, in the present cluster analysis, the subgroup of obese individuals without T2D fulfilled most of the criteria for metabolically healthy obesity [38], [39], we could still not define these individuals as metabolically healthy, because mean serum triglyceride concentration was > 1.7 mmol/l and blood pressure data were not systematically studied. This limitation of the study may stimulate further research on adipokine patterns related to metabolically healthy obesity. However, we used obese individuals with or without type 2 diabetes, which are matched for BMI, gender distribution, waist circumference, body fat mass, lipid parameters, and hsCrP as a model to distinguish adipokine clusters, which are related to impaired glucose metabolism. In this context, we find that adipokines which are associated with insulin sensitive obesity (adiponectin, progranulin, chemerin, RBP4) cluster significantly different in obese individuals with or without T2D. This further suggests an important role of these adipokines in linking obesity to its related disturbances of glucose metabolism. Noteworthy, for chemerin, we only find a trend for higher serum concentrations in T2D patients compared to obese individuals without diabetes suggesting that the subgroup size has not been sufficient to detect previously reported group differences [31]

Importantly, we need to consider that adipokine clusters are influenced by different anti-diabetic pharmacotherapies in the subgroup of obese patients with T2D. For several adipokines, sensitivity to anti-diabetic drugs has been shown. As examples, metformin has been shown to cause changes in circulating adiponectin [40], vaspin [41], [42], CTRP3 [43].

In the subgroup of obese individuals with T2D, we found a dissociation of a cluster more closely related to lipid metabolism markers and a cluster containing obesity, insulin sensitivity, glucose homeostasis, and inflammation parameters. This suggests that a subgroup of individuals with obesity and T2D are characterized by additional alterations in lipid metabolism, which may be reflected or mediated by adipokines including SFRP5, RBP4, CTRP3 and 5, omentin and adiponectin.

We found the most consistent and independent adipokine relationship between Nampt and resistin. Nampt and resistin are proinflammatory adipokines secreted from adipocytes, monocytes, and macrophages for which differential relationships in obesity and type 2 diabetes have been described [44]. However, our cluster analyses data suggest a coordinated regulation of these adipokines. On the other hand, we found that NAMPT significantly predicts T2D in multivariate regression analyses, whereas resistin is not significantly related to T2D. The absence of a relationship between resistin and T2D maybe due to a lack of differences in mean serum concentrations of this adipokine between obese individuals with and without T2D. The NAMPT – resistin cluster maybe a reflection of another underlying factor, which may more closely link NAMPT to T2D than resistin to T2D. Moreover, human monocyte expression of NAMPT is more closely related to parameters of glycemia, whereas resistin correlates more strongly with obesity (44). Other relationships detected in the entire cohort were significantly altered in subgroups of obese individuals with or without type 2 diabetes. For example relationships between vaspin and parameters of insulin sensitivity were significantly altered by the diabetes state in obese individuals. Vaspin has been identified as an adipokine with insulin-sensitizing effects [45] supporting the clustering to HOMA-IR. Our cluster analysis data are supported by previously reported correlations between vaspin and FPI or HOMA-IR [22], [46]. Another example for a strong influence of diabetes state on adipokine relationships is BMP7 and DLK1, which clustered differentially in obese patients with or without T2D. In patients with T2D, both adipokines are closely related to HOMA-IR. BMP7 is assumed to promote brown adipocyte differentiation and thermogenesis and thus, influence body weight and energy homeostasis [47], [48]. In lean and obese individuals with normal glucose homeostasis BMP7 correlates with β-cell function, as well as with fasting insulin, but not with anthropometric variables [49]. Noteworthy, BMP7 and DLK1 may be counter-regulated in brown adipocyte differentiation [50].

Our subgroup analyses may be influenced by biases such as a relatively small group size, concomitant medications and a selection bias due to recruitment strategy. The subpopulation of obese individuals without T2D has on average a lower HOMA-IR compared to the obese T2D group (50% have a HOMA-IR <5; 15% have a HOMA-IR <2.5). This selection bias may contribute to the closer relationships between insulin sensitivity parameters and BMI, % body fat and waist circumference in the obese subgroup without T2D compared to those with diagnosed T2D.

As an example, the well established association between adiponectin serum concentrations and insulin sensitivity [18], [19] was not confirmed in our cluster analyses of subgroups with or without T2D. Although we find adiponectin in the same main cluster as HOMA-IR in the entire cohort, it is most closely and significantly related to HDL-cholesterol. This finding is supported by a previous study demonstrating a significant correlation between adiponectin and HDL-cholesterol, independently of BMI and insulin sensitivity [51]. Because HDL-cholesterol is also associated with insulin sensitivity [52], the close relationship between adiponectin and HDL-cholesterol may be considered as indirect evidence for the validity of this cluster. Since we did not measure insulin sensitivity by more sensitive methods such as euglycemic-hyperinsulinemic clamps, we can not exclude that other factors that regulate both adiponectin and insulin sensitivity may have interfered with this established relationship. Fat distribution, specifically visceral obesity may represent such an underlying factor [53]. Moreover, adiponectin release from adipocytes is down-regulated under adverse metabolic conditions, resulting in decreased adiponectin serum concentrations [19]. Interestingly, various hormones associated with insulin resistance and obesity including catecholamines, insulin, glucocorticoids, TNFα and IL-6 down-regulate adiponectin expression and secretion in fat cells *in vitro* [54]. Since we did not systematically study these factors, we can not exclude an interference with the relationship between adiponectin and insulin sensitivity parameters in our analyses. The dissociation of adiponectin from the clusters related to parameters of insulin sensitivity in subcluster analyses of obesity subgroups (with or without T2D) maybe due to smaller group size and could be influenced by anti-diabetic medications or other factors. In this context, circulating adiponectin may have been altered towards unexpectedly higher concentrations by metformin treatment in the majority of obese T2D patients [40], [55].

An association of visceral fat mass with insulin resistance independent of BMI and total body fat mass has been repeatedly reported [8], [56]–[58]. Altered fat distribution may significantly affect adipokine clusters. However, further studies are required to determine the effect of visceral fat distribution on adipokine clusters. In addition, it has been shown that glucose lowering medications including metformin may affect adipokine serum concentrations [40], [43], [55], [59], [60]. We can therefore not exclude that some of the relationships among adipokines in the T2D subgroup are influenced by metformin treatment.

Independently of diabetes, glypican 4, clusterin, chemerin and progranulin are related with parameters of insulin sensitivity, suggesting that these adipokines may play a role in the development of insulin resistance. Supporting this hypothesis, we recently demonstrated that progranulin and chemerin serum concentrations are increased in patients with prediabetes (impaired fasting glucose, impaired glucose tolerance or both) compared to normal glucose tolerance individuals [3]. Moreover, increased circulating concentrations of chemerin [61] and progranulin [62], [63] are associated with insulin resistance. Both adipokines are chemoattractant proteins and contribute to recruitment of macrophages into adipose tissue [62]–[64], suggesting that inflammation of adipose tissue and alterations in cellular composition of adipose tissue contribute to the development of insulin resistance.

We further addressed the question whether adipokines or adipokine patterns may detect T2D more precisely than classical parameters. Multivariate linear regression analyses as well as PCA and PLS-DA revealed that even the combination of all 20 adipokines had a lower sensitivity (78 versus 91%) and specificity (76 versus 94%) to detect T2D compared to a combination of HbA1c, HOMA-IR and fasting plasma glucose. Importantly, our multivariate linear regression models confirm the strong effect of age in the prediction of T2D [65], suggesting the validity of these models.

However, adipokines may currently not add to the diagnostic tools for T2D. In the future, adipokines may be used to identify subgroups of individuals at risk for T2D or to early diagnose patients with insulin resistance and adipose tissue dysfunction. The clinical relevance of adipokine measurements to estimate the T2D risk has been proven for adiponectin [66]. Logistic regression analyses further revealed ANGPTL6, DLK1, Nampt and progranulin as strongest adipokine-predictors of T2D in obese individuals. Whether these adipokines predict the development of T2D needs to be tested in longitudinal epidemiological studies. The clinical use of adipokines is (at least for the more recently discovered adipokines) limited by the availability of validated and standardized assays with high sensitivities and specificities.

In conclusion, we identified distinct adipokine clusters related to fat mass and inflammation (ANGPTL3, chemerin, clusterin, DLL1, GPX3, Nampt, resistin), insulin sensitivity, glucose and lipid metabolism (adiponectin, ANGPTL6, progranulin, RBP4, BMP7, DLK1, SFRP5, vaspin, glypican4, CTRP3 and 5, omentin). Our unbiased approach identified previously unrecognized relationships among individual adipokines and between adipokines and classical parameters of obesity, inflammation and metabolic diseases, which may stimulate further systematic research to identify underlying mechanisms. An adipokine pattern consisting of ANGPTL6, DLK1, Nampt and progranulin was the strongest independent correlate of T2D in obese individuals. However, even inclusion of all 20 adipokines into a model predicting T2D had a lower sensitivity and specificity to detect T2D compared to established parameters of insulin sensitivity and glucose homeostasis.

## Supporting Information

Figure S1 **Plot of principal component analysis (PCA).** Weights of features for the first two principal components for obese patients with type 2 diabetes (T2D) and obese individuals without type 2 diabetes (no T2D).
(DOC)

Figure S2 **Plot of partial least square discriminant analysis (PLS-DA).** Weights of features for the first two principal components for obese patients with type 2 diabetes (T2D) and obese individuals without type 2 diabetes (no T2D).
(DOC)

Table S1 **Parameters of principal component analysis.** Weights of features for the first two principal components (PC1 and PC2), p value of U-Test statistics to compare features between obese patients with T2D (type 2 diabetes) and obese individuals without type 2 diabetes. Abbreviations: BMI, body mass index; HOMA-IR, Homeostatic Model Assessment – insulin resistance; FFA, free fatty acids; hsCRP, high sensitive C-reactive protein; LPS, Lipopolysacharid (Endotoxin); ANGPTL 3, angiopoietin-like protein 3; ANGPTL 6, angiopoietin-like protein 6; BMP7, bone morphogenetic protein 7; CTRP3, complement C1q tumor necrosis factor-related protein 3; CTRP5, complement C1q tumor necrosis factor-related protein 5; DLL1, delta-like protein 1; DLK1, preadipocyte factor 1; GPX3, glutathione peroxidase 3; NAMPT, nicotinamide phosphoribosyltransferase (visfatin); RBP4, retinol binding protein 4; SFRP5, secreted frizzled-related protein-5.
(DOC)

Table S2 **Parameters of partial least square discriminant analysis (PLS-DA).** VIP values (variable importance in projection) to assess the importance of features regarding discrimination of type 2 diabetes (T2D) and no T2D. Abbreviations: BMI, body mass index; HOMA-IR, Homeostatic Model Assessment – insulin resistance; FFA, free fatty acids; hsCRP, high sensitive C-reactive protein; LPS, Lipopolysacharid (Endotoxin); ANGPTL 3, angiopoietin-like protein 3; ANGPTL 6, angiopoietin-like protein 6; BMP7, bone morphogenetic protein 7; CTRP3, complement C1q tumor necrosis factor-related protein 3; CTRP5, complement C1q tumor necrosis factor-related protein 5; DLL1, delta-like protein 1; DLK1, preadipocyte factor 1; GPX3, glutathione peroxidase 3; NAMPT, nicotinamide phosphoribosyltransferase (visfatin); RBP4, retinol binding protein 4; SFRP5, secreted frizzled-related protein-5.
(DOC)
