# Supplementary material for: Identification of Adipokine Clusters Related to Parameters of Fat Mass, Insulin Sensitivity and Inflammation
Source: PLoS One. 2014 Jun 26;9(6):e99785. doi: 10.1371/journal.pone.0099785 (PMC4072672; doi:10.1371/journal.pone.0099785)
Supplement: Table S1 — Parameters of principal component analysis. Weights of features for the first two principal components (PC1 and PC2), p value of U-Test statistics to compare features between obese patients with T2D (type 2 diabetes) and obese individuals without type 2 diabetes. Abbreviations: BMI, body mass index; HOMA-IR, Homeostatic Model Assessment – insulin resistance; FFA, free fatty acids; hsCRP, high sensitive C-reactive protein; LPS, Lipopolysacharid (Endotoxin); ANGPTL 3, angiopoietin-like protein 3; ANGPTL 6, angiopoietin-like protein 6; BMP7, bone morphogenetic protein 7; CTRP3, complement C1q tumor necrosis factor-related protein 3; CTRP5, complement C1q tumor necrosis factor-related protein 5; DLL1, delta-like protein 1; DLK1, preadipocyte factor 1; GPX3, glutathione peroxidase 3; NAMPT, nicotinamide phosphoribosyltransferase (visfatin); RBP4, retinol binding protein 4; SFRP5, secreted frizzled-related protein-5. (DOC) [file pone.0099785.s003.doc]

**Table S1.** **Parameters of principal component analysis.**

| **trait** | **PC1** | **PC2** | **p-value** |
| --- | --- | --- | --- |
| gender | -0.122 | 0.375 | 0.685 |
| age | 0.113 | -0.0926 | **0.000178** |
| BMI | -0.374 | -0.103 | 0.191 |
| waist | -0.327 | -0.243 | 0.06 |
| body_fat | -0.364 | 0.162 | 0.858 |
| HbA1c | -0.0617 | -0.258 | **9.57E-23** |
| HOMA_IR | -0.094 | -0.245 | **9.64E-05** |
| TG | 0.128 | -0.37 | 0.302 |
| HDL | -0.0512 | 0.371 | 0.885 |
| FFA | -0.075 | -0.078 | 0.0727 |
| CRP | -0.133 | -0.0616 | 0.0793 |
| LPS | 0.0245 | -0.000681 | 0.905 |
| Adiponectin | -0.0709 | 0.259 | 0.816 |
| ANGPTL3 | -0.227 | 0.098 | 0.497 |
| ANGPTL6 | -0.0536 | -0.223 | **0.00458** |
| BMP7 | -0.0514 | 0.135 | 0.515 |
| Chemerin | -0.196 | -0.207 | 0.242 |
| Clusterin | -0.156 | -0.0408 | 0.697 |
| CTRP3 | 0.126 | 0.163 | 0.616 |
| CTRP5 | -0.0354 | -0.0829 | 0.52 |
| Leptin | -0.327 | 0.0603 | 0.926 |
| Glypican4 | -0.0517 | -0.0108 | 0.192 |
| GPX3 | -0.0179 | -0.0543 | 0.103 |
| DLL1 | -0.303 | -0.0304 | 0.401 |
| DLK1 | -0.0503 | 0.131 | **0.0361** |
| NAMPT | -0.25 | -0.0384 | **0.0136** |
| Omentin | 0.128 | -0.0143 | **0.0412** |
| Progranulin | -0.0641 | -0.21 | **0.00171** |
| RBP4 | 0.0506 | -0.216 | 0.626 |
| Resistin | -0.311 | 0.0519 | 0.934 |
| SFRP5 | 0.003 | -0.00355 | 0.313 |
| Vaspin | -0.0915 | 0.0282 | 0.752 |
