# Supplementary material for: Identification of Adipokine Clusters Related to Parameters of Fat Mass, Insulin Sensitivity and Inflammation
Source: PLoS One. 2014 Jun 26;9(6):e99785. doi: 10.1371/journal.pone.0099785 (PMC4072672; doi:10.1371/journal.pone.0099785)
Supplement: Table S2 — Parameters of partial least square discriminant analysis (PLS-DA). VIP values (variable importance in projection) to assess the importance of features regarding discrimination of type 2 diabetes (T2D) and no T2D. Abbreviations: BMI, body mass index; HOMA-IR, Homeostatic Model Assessment – insulin resistance; FFA, free fatty acids; hsCRP, high sensitive C-reactive protein; LPS, Lipopolysacharid (Endotoxin); ANGPTL 3, angiopoietin-like protein 3; ANGPTL 6, angiopoietin-like protein 6; BMP7, bone morphogenetic protein 7; CTRP3, complement C1q tumor necrosis factor-related protein 3; CTRP5, complement C1q tumor necrosis factor-related protein 5; DLL1, delta-like protein 1; DLK1, preadipocyte factor 1; GPX3, glutathione peroxidase 3; NAMPT, nicotinamide phosphoribosyltransferase (visfatin); RBP4, retinol binding protein 4; SFRP5, secreted frizzled-related protein-5. (DOC) [file pone.0099785.s004.doc]

**Table S2.** **Parameters of partial least square discriminant analysis (PLS-DA).**

| **trait** | **VIP1** | **VIP2** |
| --- | --- | --- |
| gender | 0.195 | 0.45 |
| age | **2.01** | **1.87** |
| BMI | 0.467 | 0.641 |
| waist | 0.637 | 0.987 |
| body_fat | 0.00744 | 0.258 |
| HbA1c | **3.9** | **3.7** |
| HOMA_IR | **1.48** | **1.37** |
| TG | 0.528 | 0.586 |
| HDL | 0.143 | 0.23 |
| FFA | 0.614 | 0.626 |
| CRP | 0.576 | 0.583 |
| LPS | 0.271 | 0.29 |
| Adiponectin | 0.219 | 0.203 |
| ANGPTL3 | 0.165 | 0.367 |
| ANGPTL6 | **1.27** | **1.26** |
| BMP7 | 0.111 | 0.14 |
| Chemerin | 0.609 | 0.738 |
| Clusterin | 0.158 | 0.307 |
| CTRP3 | 0.239 | 0.422 |
| CTRP5 | 0.435 | 0.404 |
| Leptin | 0.192 | 0.643 |
| Glypican4 | 0.646 | 0.688 |
| GPX3 | 0.589 | 0.831 |
| DLL1 | 0.253 | 0.435 |
| DLK1 | 0.819 | 0.883 |
| NAMPT | **1.28** | **1.19** |
| Omentin | 0.97 | 0.984 |
| Progranulin | **1.34** | **1.31** |
| RBP4 | 0.685 | 0.68 |
| Resistin | 0.118 | 0.131 |
| SFRP5 | 0.0416 | 0.25 |
| Vaspin | 0.3 | 0.405 |
